# Opium Alkaloids in Harvested and Thermally Processed Poppy Seeds

**DOI:** 10.3389/fchem.2020.00737

**Published:** 2020-08-27

**Authors:** Michelle G. Carlin, John R. Dean, Jennifer M. Ames

**Affiliations:** Department of Applied Sciences, Northumbria University, Newcastle upon Tyne, United Kingdom

**Keywords:** poppy seeds, morphine, codeine, thebaine, noscapine, papaverine, workplace/roadside drug testing

## Abstract

The opium alkaloids (morphine, codeine, thebaine, noscapine, and papaverine) have been detected on poppy seeds; they are widely used by the food industry for decoration and flavor but can introduce opium alkaloids into the food chain. Of the opium alkaloids found on poppy seeds, morphine, and codeine are the most pharmacologically active and have been detected in biological matrices collected in workplace and roadside drug testing resulting in positive opiate results. The European Food Safety Authority introduced an acute reference dose of 10 μg morphine/kg of body weight as a safe level for morphine in food products. In this work, it was found that in harvested poppy seeds, and thermally processed poppy seeds (with and without a food matrix), if used in normal levels would not exceed the recommended acute reference dose. It was also shown that the levels of all alkaloids reduce when thermally processed, in comparison with harvested, untreated seeds.

## Introduction

*Papaver somniferum L*. the opium poppy originated in Sumer, a region in ancient Mesopotamia (modern day Iraq and Kuwait) around 5000 BC (Aragón-Poce et al., 2002). Much has been written with respect to the opium poppy and many relate not only to the cultivation and harvest of this crop but to the investigations of the chemistry of the plant and its medicinal uses as well as the wars that have been fought over opium (Duke, 1973; Kapoor, 1995; Bernath, 1999; Miller and Tran, 2000; Aragón-Poce et al., 2002; Askitopoulou et al., 2002; Martinez-Fernández et al., 2002; Schiff, 2002; Bozan and Temelli, 2008; Cordell, 2013; Windle, 2013).

**Figure 1:**
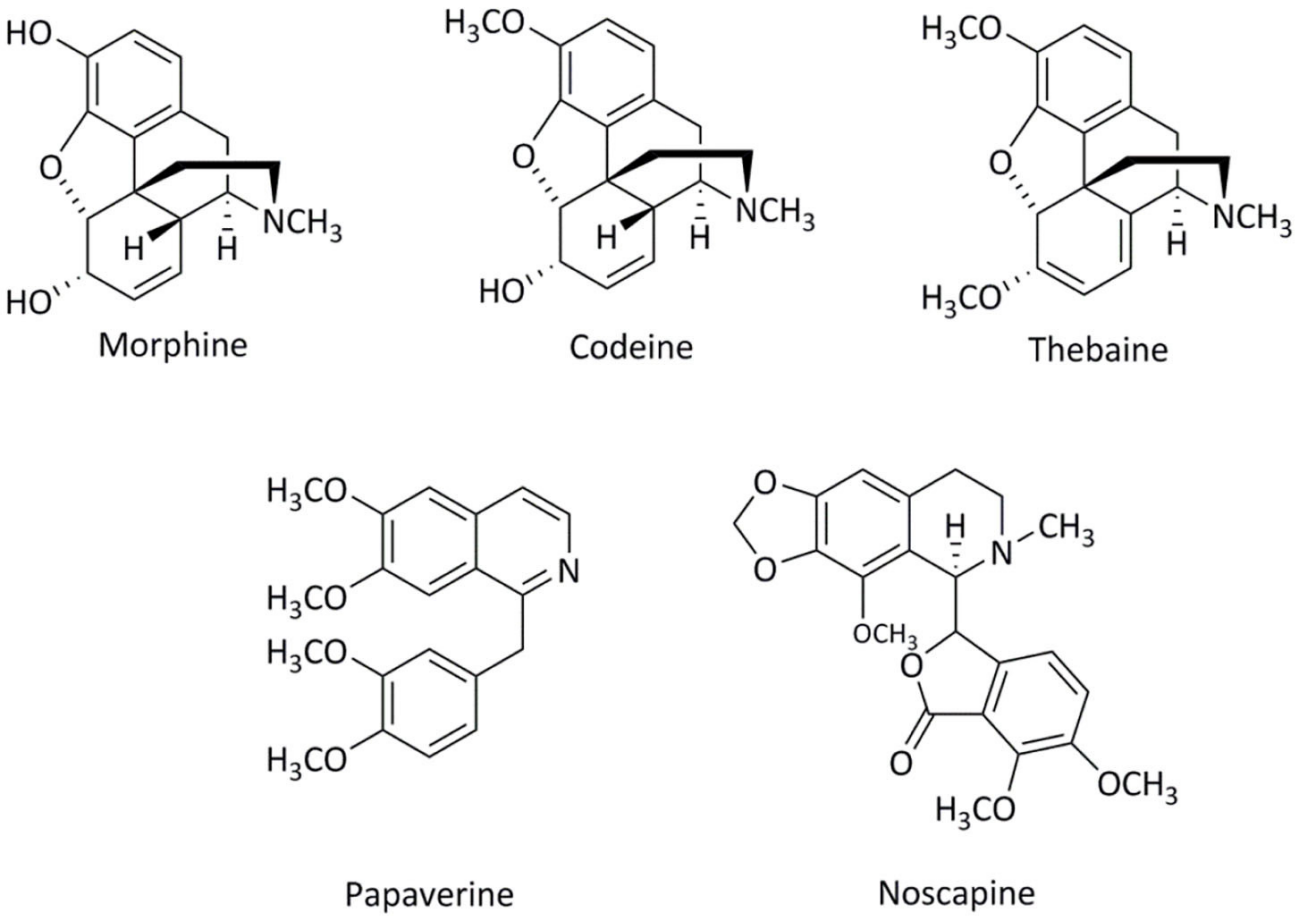
Chemical structures of the major opium alkaloids.

In the 21^st^ century, the two main legitimate uses of the opium poppy are as a source of alkaloid compounds for the pharmaceutical industry and as a source of poppy seeds for the food industry (Gumuscu et al., 2008). Of the plant family Papaveraceae (common name poppy) the genus *Papaver* has two species containing morphine, codeine, thebaine, noscapine (also called narcotine), and papaverine (Figure 1): *Papaver somniferum L*. and *Papaver setigerum D.C* (Garnock-Jones and Scholes, 1990; Yoshimatsu et al., 2005; Mohsin et al., 2012). Thebaine has been reported in *Papaver orientale L. and Papaver bracteatum* Lindl. but no biosynthetic interconversion to codeine and morphine has been found in these species (Stermitz and Rapoport, 1961). Although it is known that alkaloid compounds can be found in both *Papaver somniferum L*. and *Papaver setigerum D.C*. the former has considerably higher levels of the five major alkaloids, by percent weight, of opium than that present in *setigerum* (Table 1).

**Table 1:** Alkaloid content by % weight of opium in *Papaver setigerum* and *Papaver somniferum*.

	***Papaver subspecies***	
**Alkaloid**	Setigerum^*^	***Somniferum^+^ %***
Morphine	2.3%	7.65–25.15
Codeine	2.6%	1.21–6.37
Thebaine	Detected but not quantified	0.97–6.38
Papaverine	4.7%	0.51–5.33
Noscapine	10.2%	4.03–15.22

* as determined by electrophoresis (Panicker et al., 2007),

+ *as determined by HPLC (Krenn et al., 1998)*.

*Papaver somniferum L*. is an annual crop cultivated worldwide but is legitimately grown by the pharmaceutical and food industries in Australia, Canada, Central and Southern America, Czechoslovakia (now the Czech Republic and Slovakia), France, Holland, Hungary, India, Iran, Poland, Romania, Spain, Turkey, and (the former) Yugoslavia (Bernath, 1999; International Narcotics Board, 2016).

*Papaver somniferum L*. is an herbaceous plant which is harvested for its latex 5–10 days after the flowering petals fall from the plant (Schiff, 2002). The dried latex product is called opium from which morphine and other alkaloid compounds can be extracted. However, if left to fully mature, the plant will form poppy seeds within the capsule which can be mechanically harvested and collected by a sieving process (Kapoor, 1995; Schiff, 2002).

Harvesting methods have been shown to greatly affect the level of alkaloids in the final opium product (Mahdavi-Damghani et al., 2010). If the opium is harvested too early in the process the product is found to be watery and if too late the opium contains significantly lower levels of alkaloids (Mahdavi-Damghani et al., 2010).

*Papaver somniferum L*. is cultivated for the pharmaceutical industry but a by-product of the process of harvesting poppy straw is poppy seeds (International Narcotics Board, 2016). This source of poppy seeds is used by the food industry and are included in cakes, on bread products and sold to supermarkets and specialist shops for use in cooking/baking recipes. It was initially thought that the seeds and any products derived from them would not contain any alkaloid compounds due to the fact that the seeds develop after the latex (Duke, 1973; Schiff, 2002). However, in the late 1970's, it was noted that poppy seeds contained alkaloids found in opium (Grove et al., 1976). Meadway et al. (1998) highlighted that it was possible to fail a urine drug test after the consumption of a bread product containing poppy seeds (Meadway et al., 1998). Since then, it has become increasingly apparent that the presence of alkaloids in the food chain is a problem and can potentially lead to serious repercussions (Garnock-Jones and Scholes, 1990; Cassella et al., 1997; Bonicamp and Santana, 1998; Meadway et al., 1998; Sproll et al., 2006; Cone and Huestis, 2007; Lachenmeier et al., 2010; Chen et al., 2014; Smith et al., 2014). In Germany in particular, where poppy seeds are used and readily incorporated into food products, measures have been taken to reduce the amount of morphine present in poppy seeds intended for the food chain. It has been reported that poppy seeds used for decorative purposes can contain up to 100 mg/kg of morphine however, German authorities have recommended a limit of 20 mg/kg (Sproll et al., 2007). The European Food Safety Authority (EFSA) have published information relating to alkaloids in food products and provided a risk assessment with respect to public health (EFSA, 2011). The Report highlighted the requirement to estimate the dietary morphine exposure. Based on information provided from three European countries it was estimated that daily intake of morphine ranged from 3–90 μg/kg body weight per day. It was also hypothesized, using the same data that portions of food items having high poppy seed content could provide morphine exposure in the range 38–200 μg/kg body weight per portion for adults. From evaluation of extensive scientific literature sources, the EFSA panel concluded that it was possible for an individual to suffer effects from ingestion of poppy seed products. Importantly it has also been shown that washing, and other pre-treatments, of the seeds can reduce morphine levels by up to 90%; it is believed that the alkaloid content of poppy seeds is due to external contamination from the pod previously containing the latex and not from the inside of the seeds (Sproll et al., 2006). It is known that the ingestion of poppy seeds has caused positive opiate drug test results with positive opiate results have been found in urine, blood, and oral fluid (Cassella et al., 1997; Bonicamp and Santana, 1998; Meadway et al., 1998; Rohrig and Moore, 2003; Wong et al., 2005; Carlin, 2009; Jankovičová et al., 2009; Lachenmeier et al., 2010; Chen et al., 2014).

In this study, the aim was to establish if thermal processing methods, the food matrix employed and the source of poppy seeds would affect the levels of opium alkaloids identified pre- and post-baking. The main reason for this was to establish if normal food preparation techniques, employed when using poppy seeds, would ultimately affect the opium alkaloids reaching the food chain. Ultimately this will influence drug-testing results or have a potential clinical impact on an individual.

## Experimental

### Chemicals, Reagents and Poppy Seeds

Organic solvents (acetonitrile, chloroform, isopropyl alcohol, diethyl ether, and methanol) of HPLC grade were purchased from Sigma-Aldrich (Poole, Dorset). Deuterated morphine (100 μg/mL^−^ in methanol was purchased from Sigma Aldrich (Poole, Dorset). Liquid nitrogen was obtained from BOC Industrial Gases (Manchester). Poppy seeds were purchased from a number of retail outlets in the UK with the country of origin identified where available (Table 2).

**Table 2:** Poppy seed source and country of origin.

**Poppy seed source reference**	**Country of origin stated on label**
#1	China
#2	Unknown
#3	Turkey
#4	Unknown
#5	Holland
#6	Netherlands
#7	Unknown
#8	Netherlands

### LC-MS Instrument

HPLC was performed using an LC Surveyor system (Thermo Finnigan, Hemel Hempsted, UK) which was equipped with a pump, auto-sampler, and column heater. Mass spectrometry was performed using an LCQ advantage (Thermo Finnigan, Hemel Hempsted, UK) ion trap mass spectrometer. An Allure pentafluorophenyl phase with a propyl spacer (PFPP) column 5 μm, 50 × 2.1 mm fitted with an Allure PFPP 10.0 × 2.1 mm guard column (both Restek, Buckinghamshire, UK) were employed. The HPLC was run using a gradient method (Table 3) with the column thermostated to 40°C and the autosampler tray held at 8°C.

**Table 3:** Mobile phase composition and gradient program for HPLC.

**Mobile phase composition**			
**Solvent A:** Water + 2 mM ammonium formate + 0.2% formic acid, pH 2.4			
**Solvent B:** Acetonitrile + 2 mM ammonium formate + 0.2% formic acid, pH4.8			
**Time (mins)**	**%A**	**%B**	**Flow rate (μL/min)**
0.00	90	10	350
2.00	90	10	350
10.00	10	90	350
11.00	10	90	350
12.00	90	10	350
14.00	90	10	350

The mass spectrometer was operated in positive mode ionization with the specific instrument parameters shown in Table 4 (Carlin et al., 2017).

**Table 4:** Analyte specific parameters for LCQ Advantage mass spectrometer from HPLC.

**Compound**	***m/z***	**Monitored transition mass *(m/z)***	**Collision Energy (eV)**
Morphine	286	286 → 201, 229	33
Morphine-d3	289	289 → 201, 229	30
Codeine	300	300 → 215, 243, 282	32
Thebaine	312	312 → 183, 249, 281	28
Papaverine	340	340 → 202	36
		202 → 171	32
Noscapine	414	414 → 220, 353	29

**Figure 2:**
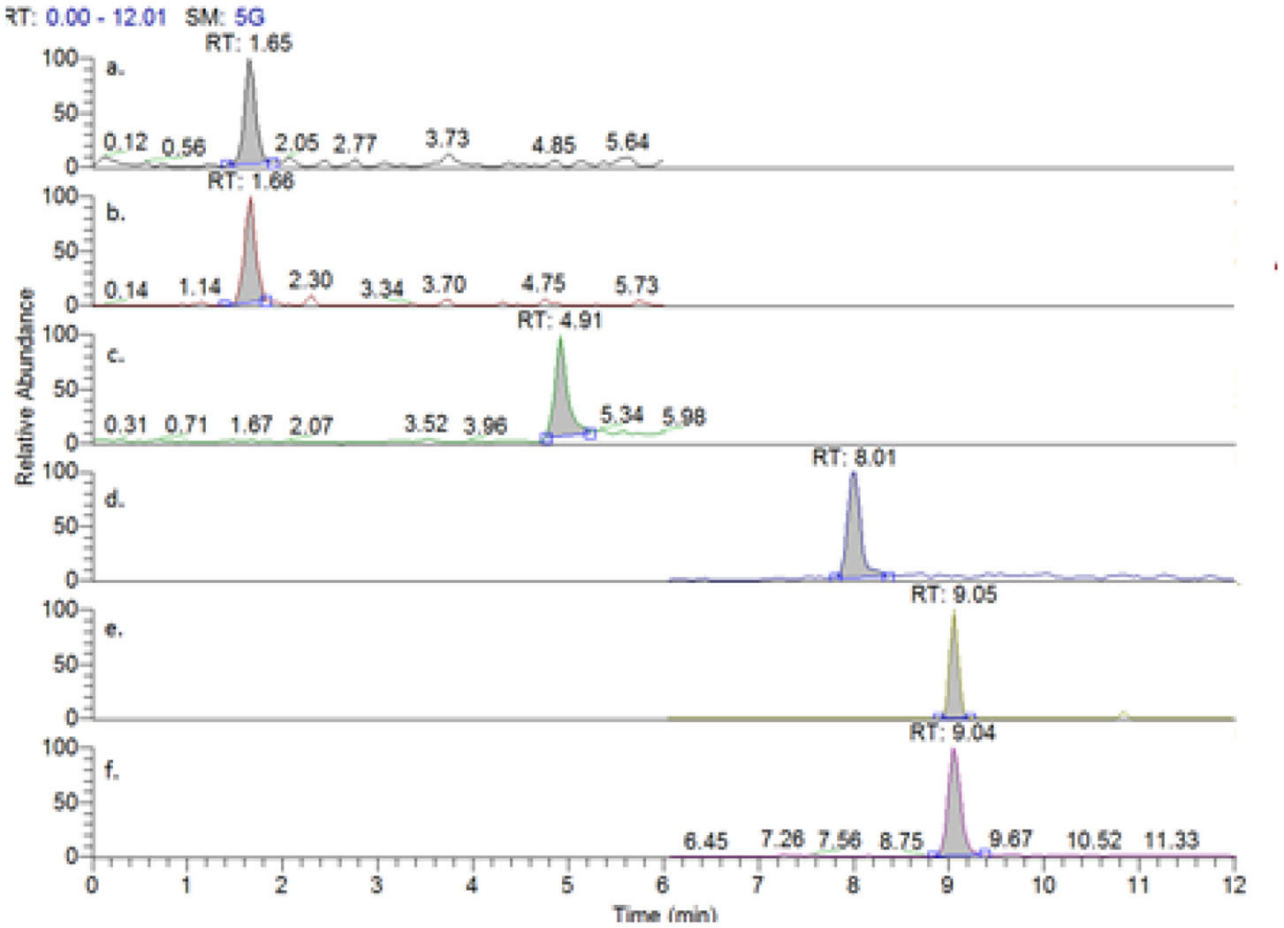
Extracted chromatograms from a mixed injection of (a) morphine, (b) morphine-d3, (c) codeine, (d) thebaine, (e) papaverine, and (f) noscapine.

Figure 2 shows the extracted ion chromatograms for each of the alkaloids being analyzed in this work, including deuterated morphine as an internal standard. An overall run time of 12 min was employed, with all analytes of interest eluting before 10 min.

### Method Validation

In the validation of the LC-MS method, calibration models were prepared by using the concentration versus the ratio of the drug peak area/internal standard peak area. A minimum of six calibration points, plus a blank, were used with the concentration range and associated linear equations and *R*^2^ values (Table 5). A calibration set was analyzed alongside every data set. The limit of detection (LOD) for this method was determined to be <2 ng/mL and the limit of quantitation (LOQ) was determined to be 10 ng/mL. Precision and accuracy were found to be less than 6% for all alkaloids. Analyte specific validation data is shown in Table 5.

**Table 5:** Validation parameters for morphine, codeine, thebaine, papaverine, and noscapine.

**Compound**	**Linear equation**	**R^**2**^**	**Concentration range**	**LOQ**	**LOD**	**Precision**	**Repeatability**
			**(ng/mL)**	**(ng/mL)**	**(ng/mL)**	**%CV**	**%CV**
Morphine	y = 0.0047x−0.0100	0.9957	0–200	10	1.5	1.1	2.9
Codeine	y = 0.014x−0.0675	0.9976	0–200	10	2	2.7	4.8
Thebaine	y = 0.0214x−0.2152	0.9915	0–200	10	2	1.5	4.5
Papaverine	y = 0.019x−0.1795	0.9952	0–400	10	2	1.8	5.8
Noscapine	y = 0.0735x−0.6081	0.9985	0 – 500	10	1.5	3.6	4.6

### Alkaloid Extraction From Poppy Seeds

Seven solvents, which covered a broad range of polarity index values (Table 6) and a solvent mix, chloroform: isopropanol (90:10, v/v), were investigated with respect to alkaloid extraction efficiency.

**Table 6:** Extraction solvents and associated polarity index values.

**Solvent**	**Polarity index (snyder)^1^**
Diethyl ether	2.8
Dichloromethane	3.1
Chloroform	4.1
Isopropanol	4.3
Acetonitrile	5.8
Methanol	6.6
Water	9
Chloroform/isopropanol (90:10, v/v)	Unknown

1 *Snyder's Polarity Index Values of Common Laboratory Solvents. Available online at: http://www.sanderkok.com/techniques/hplc/eluotropic_series_extended.html#1*.

For each solvent, extractions were carried out at a different pH's (i.e., pH 3.5, pH 5.0, pH 7.0, and pH 9.0) to obtain the optimum extraction conditions for the alkaloids from the poppy seeds. To avoid contamination by plastic residues from pipette tips by the solvents, glass pipettes were employed.

Poppy seeds from a portion of the #1 seeds were homogenized using a spice blender and approximately 200 mg of the seeds were weighed into glass Durham tubes (four tubes for each of the solvents). For each solvent, the pH was altered to produce a solution of poppy seeds and solvent (1 mL) at the specified pHs. To each solution, deuterated internal standard (morphine-d_3_) was added. The tubes were then capped and placed into an ultrasonic bath for 10 min, centrifuged at 4,000 rpm for 10 min. The appropriate layer was transferred to a clean glass tube, where it was dried down under nitrogen at 40°C and reconstituted in 100 μL of aqueous mobile phase: extracts were filtered using a 0.22 μm nylon syringe filter before being transferred to a glass insert held in an auto-sampler vial for the LC-MS instrument.

Calibration solutions were prepared in the mobile phase on the day of analysis for each of the extractions. The resultant equations from the calibration were used to determine the concentration of the solution of poppy seeds; the final alkaloid levels in ng/g were determined by taking into account the dilution factor from the solvent and the original weight of the poppy seeds used. The reason that calibration curves were produced on each analysis day was that it was shown in previous extractions, that small sub-samples of poppy seeds from the same packet/batch showed much variation of the concentration of the alkaloids present. This is in keeping with the findings of other researchers, who identified a variation in opium alkaloids within a batch and between batches of poppy seeds analyzed (Bozan and Temelli, 2008; López et al., 2018).

### Preparations of Poppy Seeds and Bakery Products

#### Poppy Seed Muffins

Mini poppy seed muffins were prepared by mixing together 175 g of self-raising flour, 112 g of caster sugar, 50 g of poppy seeds, ½ teaspoon of bicarbonate of soda, 70 g of melted butter, two small eggs, the zest of one lemon, and 175 mL of skimmed milk. The ingredients were mixed into a batter and added to each of the dimples of mini-muffin trays purchased from Lakeland (Ambleside, Cumbria). The trays were then placed into an electric oven set to 180°C and left to cook for 15 min. The final weight of poppy seeds in each mini-muffin was approximately 1.8 g. They were then left to cool to room temperature. Each poppy seed muffin weighed ~20 g. The poppy seed muffins were immersed in liquid nitrogen, crushed using a mortar and pestle and transferred to a spice blender for homogenisation prior to extraction and analysis by LC-MS. The LC-MS method employed has been previously reported in the literature (Carlin et al., 2017). The liquid nitrogen method was found to be easiest to apply to the muffins: this was due to the fact that the poppy seeds were incorporated into the sponge of the muffin. Trying to extract each poppy seed from the matrix proved very time consuming and was also considered that any alkaloids that may have interacted with the muffin matrix may also be included in results. Less fatty emulsion was also formed during the extraction method, when liquid nitrogen was employed.

#### Poppy Seed Topped Rolls

A comparative study was carried out to establish if there was a difference between alkaloid levels resulting from poppy seeds incorporated into the matrix of the muffin to those resulting from poppy seeds coated onto a bread roll. The dough for the rolls was prepared using 280 g of strong white bread flour, 1½ tablespoons of sugar, one teaspoon of salt, ¾ teaspoon of fast action yeast, two tablespoons of skimmed milk powder, 150 mL of water, and two tablespoons of oil. The dough was mixed in a commercially available Morphy Richards compact bread maker (using the “dough” program). The dough was then split into 4 equal portions and each one was pressed into poppy seeds. The rolls were then placed into an oven at 190°C and left to cook for 25 min, according to the recipe of the bread mix. The rolls were left to cool to room temperature and the poppy seeds were scraped from the surface using a metal spatula and homogenized in a spice blender prior to extraction and analysis by LC-MS.

#### Thermally Processed Poppy Seeds

In order to assess if the muffin matrix had any effect on the level of alkaloids found in the poppy seeds, raw poppy seeds from different suppliers were placed on a baking tray and heating at 180°C for 15 min: they were then left to cool to room temperature, homogenized in a spice blender prior to extraction and analysis by LC-MS.

## Results and Discussion

### Extraction Method

The resulting chromatograms from the poppy seed extractions were obtained for each of the alkaloid compounds and were compared with respect to the presence of alkaloid and internal standard peaks, peak shape, and interferences from the matrix/solvent combination. It was found that at the extremes of the polarity scale (diethyl ether, dichloromethane, and water), the chromatograms produced were complex and with poor peak shape for the alkaloids; these observations were independent of pH.

In contrast, the optimum result, in terms of alkaloid presence and peak shape, was obtained using the solvent mixture i.e., chloroform: isopropanol (90:10, v/v) at pH 3.5. For this reason, this extraction solvent mixture was used for all subsequent extraction's.

### Harvested Poppy Seeds

From each batch of poppy seeds, a minimum of six different portions (weighing ~200 mg) were extracted and analyzed. For each source, the mean weight of each of the alkaloids in poppy seeds was calculated. When the levels of morphine in poppy seeds from each of the different sources was compared (Table 7), it was found that there was much variation within batch but also between sources of poppy seeds. For example, no morphine was identified in any of the 15 randomly selected portions of seeds from source #4 however from source #2, when 15 randomly selected portions of these seeds were analyzed, the levels of morphine ranged from 2,638–63,994 ng/g. The range is provided to show the extent of the variation and is in keeping with other publications of this nature (López et al., 2018). There is much variation in the extracted opiate compounds, which is primarily due to the environmental differences of the seeds (Katrine et al., 2018; Lahiri et al., 2018). The country of origin for both of these poppy seed sources is unknown.

**Table 7:** Range and mean weight of alkaloids (ng/g) in poppy seeds.

	**Morphine**		**Codeine**		**Thebaine**		**Noscapine**	
**Poppy seed source reference**	**Mean weight (ng/g)**	**Range (ng/g)**	**Mean weight (ng/g)**	**Range (ng/g)**	**Mean weight (ng/g)**	**Range (ng/g)**	**Mean weight (ng/g)**	**Range (ng/g)**
#1	1233	233–3,197	2,308	1,426–4,520	1,251	285–2,480	ND	ND
#2	29,652	2,638–63,994	8,507	474–23,307	42,950	1,977–133,493	ND	ND
#3	121	ND−769	157	ND−651	ND	ND	ND	ND
#4	ND	ND	72	52–106	ND	ND	ND	ND
#5	5,840	864–10,837	2,610	ND−5,441	6,363	841–12,561	534	ND−2,970
#6	1,620	141–4,223	153	61–349	112	ND−343	ND	ND
#7	1,059	ND−4,754	5,688	236–14,607	ND	ND	2,224	291–10,700
#8	62	ND−312	117	94–157	ND	ND	ND	ND

*ND, not detected*.

When the same comparison was carried out for codeine (Table 7) it was also found that there was much variation within different portions of the same batch and between sources of poppy seeds as was the case with morphine. Source #2, which was found to have a level of morphine much higher than the other sources, was also found to have a higher level of codeine. No other similarities can be drawn from the data. When this same comparison was carried out for thebaine (Table 7) it was found that of the poppy seeds analyzed, 50% of the source seeds did not contain thebaine. It was also found that the same source with the highest levels of morphine and codeine also exhibited the highest levels of thebaine.

Noscapine was identified in only two of the eight sources of poppy seeds (Table 7). It was found that the seeds from source #7 contained the highest levels of noscapine of the two sources where noscapine was identified. When the levels of the other alkaloids present in source #7 seeds, it was found that morphine (ND−4,754 ng/g) and codeine (2,361–14,607 ng/g) were also identified at levels in the higher range with the respect to the other sources. Papaverine was detected in some of the analyzed seeds but peaks were so small that it was not possible to quantify them. Source #2 was found to contain morphine (2,638–63,994 ng/g), codeine (474–23,307 ng/g) and thebaine (1,977–133,493 ng/g) at levels higher in comparison to other sources. It has been identified that sub-varieties of *Papaver somniferum L*. will have different alkaloid content and compositions (EFSA, 2011; Stranska et al., 2013). However, this taxonomic information was not available from the suppliers of the seeds.

It has been known since 1920 (Annett, 1920) that factors, such as the season in which the plants are grown, weather conditions, and quality and type of fertilizer used can greatly affect the levels of alkaloids biosynthesised by *Papaver somniferum L*. In turn, the levels of alkaloids found in opium latex will also be affected. No data currently exist that compares levels of alkaloids in opium latex and alkaloids from the same plant but it is assumed that the levels would correlate. On this basis, the country of origin, where the plant was grown in the field (e.g., in the shade or direct sunlight) and the quality of the soil can all affect the levels of alkaloids in the poppy seeds (Moeller et al., 2004; Sproll et al., 2006). This means that if a batch of poppy seeds is harvested from one field, naturally there will be variation in the levels of alkaloids from each of the plants. It has also been shown that the alkaloids present in the opium latex may contaminate the poppy seeds as part of the growing process and that a batch of poppy seeds is the combination of multiple fields in one country: all of these factors may explain why there is such variation within batch and between sources of poppy seeds.

### Harvested Versus Thermally Processed Poppy Seeds

**Figure 3:**
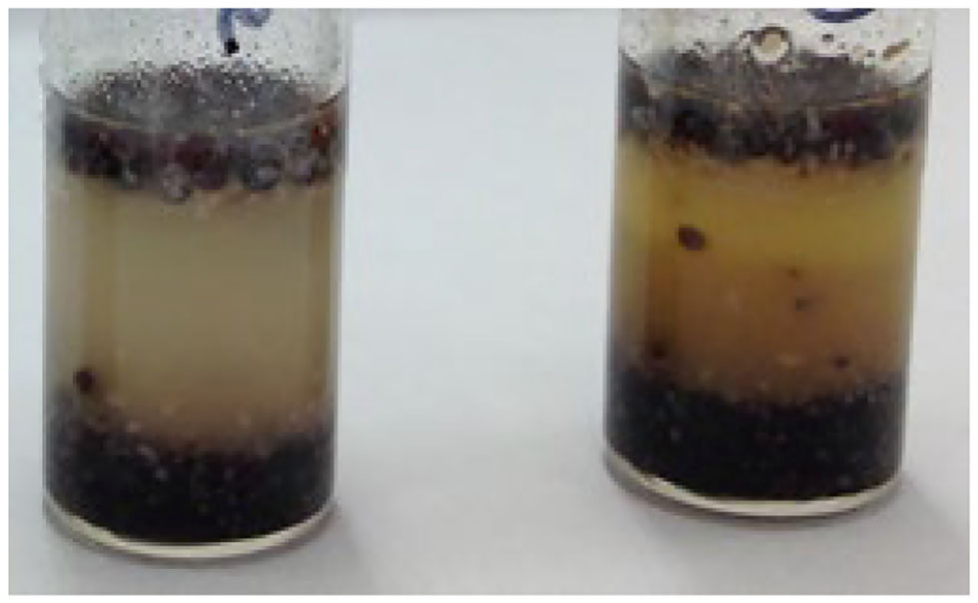
Sample tubes containing poppy seed muffin and extraction solvent, post agitation, and centrifugation.

A comparison was carried out to establish if there was a difference in the levels of alkaloids identified between harvested poppy seeds, as described above, poppy seeds which were baked on top of a bread roll, poppy seeds incorporated into a muffin matrix, and poppy seeds heated in an oven in the absence of bread/muffin matrix. For each supermarket source of harvested poppy seeds, poppy seeds heated without matrix, and poppy seeds scraped from the bread roll, 15 randomly selected portions from each packet weighing ~200 mg were extracted and analyzed while for the poppy seed muffins, ~400 mg of homogenized poppy seed/muffin mixture was extracted. However, the muffin matrix greatly interfered with the extraction process. During the extraction process, a fatty emulsion was formed which affected further sample preparation techniques (Figure 3). These aliquots were filtered twice prior to being transferred into HPLC vials however when the chromatograms were analyzed for these muffin extractions, no alkaloids were identified. For this reason, it was not possible to include the poppy seed muffins extract results in the comparison between harvested poppy seeds, thermally processed seeds on their own and poppy seeds on the top of bread buns. In addition, seed portions from three randomly selected sources were extracted and analyzed with the results shown in Table 8. Again, as was established with extractions of harvested poppy seeds there was much variation in the alkaloids identified and in the levels of those alkaloids present, Deuterated morphine was added prior to extraction of the alkaloids from the seeds and percentage extractions were incorporated into the calculations.

**Table 8:** Comparison of levels of alkaloids identified in harvested poppy seeds, seeds from the surface of bread rolls and seeds heated with no matrix.

		**Harvested**		**Seeds on bread roll**		**Heated (no matrix)**	
**Source reference**	**Alkaloid**	**Mean (ng/g)**	**Range (ng/g)**	**Mean (ng/g)**	**Range (ng/g)**	**Mean (ng/g)**	**Range (ng/g)**
#1	Morphine	545	8–1,888	11	ND−33	63	ND−304
	Codeine	82	ND−284	3	ND−27	5	ND−54
#6	Morphine	217	ND−431	1	ND−11	ND	ND
	Codeine	175	ND−418	3	ND−21	ND	ND
	Papaverine	11	ND−64	ND	ND	ND	ND
	Noscapine	34	ND−80	ND	ND	ND	ND
#8	Morphine	25	ND−96	ND	ND	15	ND−49
	Codeine	30	ND−81	5	ND - 19	11	ND−52

*ND, not detected*.

What was identified from this data was that whether the seeds were heated on the surface of the bread roll or were heated with no bread matrix, the levels of alkaloids (if detected) were considerably lower than in the harvested seeds. Koleva et al. (2012) reported that morphine content could be reduced by 10–50% in the process of baking while Sproll et al. (2007) reported that the process of grinding and baking could reduce the morphine content of poppy seeds by up to 84%.

When comparing the results from the current work to levels published in the literature (Table 9) it can be seen that these findings are in-keeping with those published by Sproll et al. (2006) however, their maximum concentrations for each of the alkaloids compared, was found to be higher than in the current study. This group of researchers employed an LC/MS/MS method for the detection of morphine, codeine, papaverine, and noscapine in poppy seeds. The work published by Grove et al. (1976) appears to show much lower levels for morphine and codeine and only these two alkaloids were included in this study. However, this could have been due to the sensitivity of the GC-MS instrument employed, the lack of information regarding the presence of other alkaloids present in poppy seeds at this time as the work reported by Grove et al. was published in 1976 when analytical instruments, such as GC-MS were not as sensitive as they are today.

**Table 9:** Comparison of alkaloids identified in poppy seeds.

	**Current work**	**Grove et al. (1976)**	**Sproll et al. (2006)**
**Compound**	**Range (ng/g)**		
Morphine	ND−63,994	600–2,300	1,000–270,000
Codeine	ND−23,307	100–500	ND−56,000
Thebaine	ND−133,493	NI	NI
Papaverine	ND	NI	ND
Noscapine	ND−10,700	NI	ND−21,000

*ND, not detected; NI, not included in the study*.

The levels of alkaloids identified in the current work are generally lower than those found by Sproll et al. (2006) but this could be due to the same factors that may influence the levels of alkaloids in poppy seeds previously highlighted. This research has shown that alkaloid variation exists depends on the specific alkaloids, their source and thermal processing. It was clear from the data obtained in this current work, and from other studies published in the literature, that there is much variation in the levels of alkaloids identified in poppy seeds. This variation can be attributed to a variety of natural parameters, such as weather and soil conditions, but also in the way that the seeds are harvested (Lachenmeier et al., 2010). Processing methods prior to packaging and even the baking process has been shown to greatly affect the level of alkaloids (Sproll et al., 2007). The findings of this study correlate with the studies published in the literature.

When poppy seeds are consumed on a bun or roll, it has been estimated that each roll contains 1–4 g of poppy seeds (Lachenmeier et al., 2010). Due to the reduction of the levels of alkaloids in the baking process, it has been estimated that if the poppy seeds contained 100,000 ng/g of morphine, the amount of seeds ingested would not cause any significant effect on an individual (Sproll et al., 2006).

Assuming that the average salad contains 3–6 g (1–2 teaspoons) of poppy seeds and the average bread bun has between 1.5–1.8 g of poppy. In addition, if 3–6 g of heated (“toasted”) poppy seeds would be used in a salad, the following values were calculated (based on the data obtained from Table 8 and using the highest value of each alkaloid determined).

The EFSA Panel on Contaminants in the Food Chain defined an acute reference dose (ARfD) of 10 μg morphine/kg (10,000 ng morphine/kg) of body weight (EFSA, 2011). If one assumes a 70 kg body weight of an “average” adult, it is possible to safely ingest 700 μg (700,000 ng) of morphine. This value cannot be used when relating to young children, the elderly or individuals of poor health (Sproll et al., 2006). With respect to the values of morphine obtained in this work for harvested seeds, seeds on top of a bread roll and seeds heated with no matrix (Table 10) and taking into account the weights of poppy seeds used in a variety of food products mentioned above, the morphine ingested will not exceed the ARfD determined by the EFSA.

**Table 10:** Comparison of alkaloids identified on harvested and thermally processed poppy seeds.

		**Harvested**	**Seeds on bread roll**	**Heated (no matrix)**
**Source reference**	**Alkaloid**	**(ng)**	**(ng)**	**(ng)**
#1	Morphine	5,666–11,330	49.5–59.4	912–1824
	Codeine	850–1700	40.5–48.6	324–648
#6	Morphine	1290–2590	16.5–19.8	–
	Codeine	1250–2500	31.5–37.8	–
	Papaverine	192–380	–	–
	Noscapine	240–480	–	–
#8	Morphine	290–580	–	147–294
	Codeine	240–490	28.5–34.2	156–312

Recently, the EFSA published an update on these guidelines (Katrine et al., 2018). This update related to the detection of morphine, codeine, oripavine, noscapine, and papaverine in poppy seed samples whereas, the previous report related only to the levels of morphine entering the food chain. Codeine values were given in relation to morphine equivalents, using a conversion factor of 0.2. Noscapine and papaverine were considered in the most recent publication however, the data that was available to the EFSA did not allow for a hazard characterization but they did conclude that the presence of these compounds would not present a health concern. In relation to the presence of thebaine and oripavine (not included in the work of this paper), it was concluded that there was insufficient data to make any assessment. Based on these updated EFSA findings, the presence of the morphine and codeine in the poppy seeds analyzed in this work, would still would still fall below the recommendations outlined.

Currently, there are no guidelines on how poppy seeds entering the food chain should be treated prior to use (López et al., 2018). There is also little or no information on packaging of poppy seeds regarding what, if any, treatment has taken place prior to packaging. Since the ingestion of poppy seeds has been used as reasons for failure of workplace drug testing and roadside drug testing, more should be done to ensure as much information as is possible is available on the preparation methods of the seeds.

## Data Availability Statement

The datasets generated for this study are available on request to the corresponding author.

## Ethics Statement

This work was approved by the Northumbria University Ethics committee. The university holds a UK Home Office Drug License for the storage and use of controlled drug standards and for the extraction of alkaloids from poppy seeds.

## Author Contributions

The laboratory work, analysis of data and writing was carried out predominantly by MC. JD and JA helped in the design and review of data and interpretation and all parties contributed to the writing and review of the article. All authors contributed to the article and approved the submitted version.

## Conflict of Interest

The authors declare that the research was conducted in the absence of any commercial or financial relationships that could be construed as a potential conflict of interest.
